# Oropouche fever cases in Salvador, Bahia, Brazil, 2024

**DOI:** 10.1016/j.bjid.2026.105806

**Published:** 2026-03-27

**Authors:** Abevailton Miranda, Róger Jesus Costa, Juan P. Aguilar Ticona, Carlos Brites, Wei Kung Wang, Eduardo Martins Netto, Centro de Pesquisa, Aprendizagem e Inovação ‒ Fundação José Silveira

**Affiliations:** a Universidade Federal da Bahia, Programa de Pós-Graduação em Medicina e Saúde, Salvador, BA, Brazil; b Secretaria de Saúde do Estado da Bahia, Salvador, BA, Brazil; c Universidade Federal da Bahia, Instituto de Saúde Coletiva, Salvador, BA, Brazil; d Ministério da Saúde, Fundação Oswaldo Cruz, Instituto Gonçalo Moniz, Salvador, BA, Brazil; e Universidade Federal da Bahia, Programa de Pós-Graduação em Microbiologia, Salvador, BA, Brazil; f Fundação Bahiana de Infectologia, Salvador, BA, Brazil; g University of Hawaii at Manoa, John A. Burns School of Medicine Department of Tropical Medicine, Medical Microbiology and Pharmacology, Honolulu, Hawaii, USA; h Fundação José Silveira, Centro de Pesquisa, Aprendizagem e Inovação, Salvador, BA, Brazil

**Keywords:** Oropouche virus, Epidemiology, Arboviruses

## Abstract

**Background:**

Oropouche Virus (OROV) is an arbovirus endemic to parts of Central and South America, historically confined to the Amazon region. In 2023‒2024, a significant expansion of OROV circulation was reported in Brazil, including autochthonous transmission in Salvador, Bahia. Despite its growing relevance, clinical descriptions are limited and mainly associated with the new lineage.

**Method:**

We conducted a passive surveillance study based on medical records of patients confirmed by RT-qPCR for OROV infection who were treated at an Emergency Care Unit in Salvador, Bahia, between April and July 2024. Sociodemographic, clinical, and laboratory data were collected.

**Results:**

We collected information from 11 RT-PCR-confirmed patients with a mean age of 38-years, and a female predominance (55 %). The most prevalent symptoms were fever (100 %) and headache (100 %), myalgia (90 %), nausea (73 %), and arthralgia (55 %). No cases of severe or fatal outcomes were observed.

**Conclusions:**

This study contributes to the clinical characterization of Oropouche fever in an emerging urban transmission setting outside the traditional endemic areas. The findings support the need for including OROV in the differential diagnosis of acute febrile illnesses, strengthening molecular surveillance, and expanding awareness among clinicians in non-Amazonian regions.

## Introduction

Oropouche fever is an arboviral disease caused by Oropouche Virus (OROV), febrile arbovirus, endemic to Central and South American countries.^1^ *Culicoides paraensis* midges have been considered the main vector of OROV in the urban cycle of the disease in the neotropics.^2^^,^^3^ It is known for causing outbreaks in the Amazon region where environmental conditions favor vector proliferation.^4^^,^^5^ Between 2023 and 2024, the virus re-emerged and expanded geographically, with reported cases in previously non-endemic areas in Brazil.^6^^,^^7^

The diagnosis of Oropouche fever relies on both clinical and laboratory criteria. However, its clinical presentation often mimics other arboviral infections such as Dengue, Zika, Chikungunya, and Yellow Fever, making clinical diagnosis challenging.^8^ Cases of aseptic meningitis with signs of meningeal irritation, hemorrhagic manifestations, and encephalitis has been documented, including instances of rapid progression from symptom onset to death in Salvador, Brazil.^9^ Moreover, the emergence of a new OROV BR-2015‒2024 clade, responsible for the epidemic in 2023‒24 may be associated with viral transmission and virulence, potentially further complicating differential diagnosis.^10^^,^^11^

With the increasing burden of emerging and reemerging arboviruses, public health surveillance is essential for early detection and response. Variations in clinical presentation hinder accurate assessment of transmission impact, underscoring the need for studies that improve diagnostic accuracy and raise clinician awareness of this infection in differential diagnoses. The first cases of Oropouche fever in Salvador were identified in 2020.^12^ In 2024, passive epidemiological surveillance detected 16 confirmed cases in the city, of which 12 were attributed to autochthonous transmission.^13^ We aimed to report the clinical manifestations observed in patients diagnosed with Oropouche fever at an Emergency Care Unit in Salvador, characterizing their epidemiological and clinical profiles.

## Methods

We conducted a passive surveillance study at an Emergency Care Unit (UPA ‒ *Unidade de Pronto Atendimento*) in Salvador, Bahia. Participants were recruited from April to July 2024. The objective was to identify arboviral infections in patients presenting with acute symptoms within one to five days after symptom onset.

The clinical case definition required the presence of fever accompanied by at least two signs or symptoms, or alternatively, three or more signs or symptoms in the absence of fever. The spectrum of clinical manifestations included headache, rash, asthenia, dizziness, conjunctivitis, cough, odynophagia, nausea, vomiting, diarrhea, epigastric pain, back pain associated with coughing, arthritis, severe arthralgia, myalgia, oral bleeding, retro-orbital pain, and petechiae. Laboratory findings encompassed leukopenia (defined as a white blood cell count <4000 mcL) and a positive tourniquet test (capillary fragility test, >15 petechiae within a 5 cm diameter circle were considerate positive).

Sociodemographic, clinical, and laboratory data were extracted using a standardized form. Information on comorbidities such as hematological diseases, liver disease, renal disease, and hypertension was also collected.

Laboratory confirmation for Oropouche Virus (OROV), dengue virus serotypes 1–4, Chikungunya virus, Zika virus, and West Nile virus infections. RNA was extracted from serum samples (100 μL) using the Loccus Extracta® RNA and Viral DNA Kit (MVXA-PU16 FAST), according to the manufacturer’s instructions. For dengue virus serotypes 1–4, Chikungunya virus, and Zika virus, a multiplex Real-Time Reverse Transcription Polymerase Chain Reaction (RT-qPCR) protocol was employed. In contrast, OROV and West Nile virus infections were confirmed using an in-house singleplex RT-qPCR assay. This assay was specifically optimized for the detection of OROV during the first five days of the acute phase of illness, ensuring sensitivity in the early stages of infection.

Laboratory testing for OROV was conducted at the Central Public Health Laboratory of Bahia (LACEN-BA). Serum samples collected from suspected cases at healthcare facilities were transported to the reference laboratory following routine surveillance procedures. Molecular detection was performed using an in-house real-time RT-PCR assay validated by the state public health laboratory. Results were interpreted qualitatively as “detectable” or “non-detectable” based on reference standards, rther details of the molecular diagnostic laboratory protocol are provided in Supplementary Material 1. Laboratory confirmation was integrated into the state arboviral surveillance program.

This study was approved by the local Research Ethics Committee (Comitê de Ética em Pesquisa da Maternidade Climério de Oliveira/UFBA, Brazil (CAAE: 25.336.819.3. 0000.5543/4.691.233, 2019).

## Results

During the study period, 54,025 patients were admitted to the emergency unit. Among them, 873 (1.6 %) were clinically diagnosed with febrile syndrome. Of these, 359 (41.1 %) met the clinical case definition and were included for molecular testing. Among those tested, 11 individuals (3.1 %) were confirmed positive for OROV infection. All samples were also tested for dengue virus serotypes 1–4, chikungunya virus, Zika virus, and West Nile virus, with all results negative.

The mean age of patients with OROV infection was 38-years (Range: 22‒58), with a predominance of females (55 %). All patients were presented with fever and headache. Other frequent symptoms included myalgia (90 %), nausea (73 %), and arthralgia (55 %). Retro-orbital pain (45 %), diarrhea (45 %), and vomiting (25 %) were also common. The mean duration of symptoms was 3-days. Signs and symptoms observed in fewer than 10 % of cases included asthenia, cough, epigastric pain, rash, lower back pain, conjunctivitis, arthritis, and dizziness. Neither leukopenia nor a positive tourniquet test was detected in any case. Importantly, no severe neurological or hemorrhagic complications were observed. Detailed clinical summaries for each case are provided in the Supplementary Material 2.

## Discussion

In our study, 3 % of participants seeking medical care tested positive for Oropouche Virus (OROV). These cases were not captured by routine passive public surveillance, underscoring that the true burden of disease is likely underestimated. All OROV-positive participants presented only mild and self-limited forms of illness, with no severe clinical manifestations observed, and no coinfections with other arboviruses were detected. When considered alongside the 16 cases detected through passive surveillance, it is plausible that asymptomatic or mild infections went unreported, further masking the actual extent of transmission.

This local picture contrasts with the broader national scenario. In 2023–2024, Brazil experienced a major OROV epidemic, marked by expansion into non-endemic regions beyond the Amazon Basin. In 2024, 13,856 cases were reported nationwide, including 904 in Bahia.^6^ Public health alerts were triggered not only by the scale of transmission but also by reports of fatalities and congenital abnormalities linked to vertical transmission.^9^^,^^14^ The unusual geographic expansion and diverse clinical outcomes may be explained by multiple exports of OROV from northern Brazil to other regions, facilitating autochthonous transmission chains. Moreover, the accumulation of mutations with potential phenotypic effects raises concerns about changes in the viral transmissibility and virulence.^15^ In our study no severe cases associated with OROV infection were identified, and all infections presented as mild and self-limited. On the other hand, the 3 % incidence observed among participants ‒ compared with the 16 cases detected through passive surveillance in Salvador (corresponding to an incidence of approximately 0.6 per 100,000 inhabitants) ‒ suggests that the true infection rate is underestimated. This aligns with the hypothesis of increased transmissibility, although other factors must also be considered. Ecological and environmental variations may have favored transmission, including possible vector competition.^11^ While *C. paraensis* is widely disseminated across Brazil, other vectors may also contribute to OROV transmission.^16^^,^^17^

Surveillance capacity and access to healthcare vary substantially across regions. Historically, OROV-endemic areas have had weaker surveillance systems and limited healthcare access, contributing to systematic underestimation of the true infection burden. Furthermore, the guidelines in 2024 limit OROV testing to just 10 % of arbovirus-suspected samples negative for Zika, Dengue, and Chikungunya. In our study, all identified cases presented as mild, self-limited febrile illness accompanied by systemic and gastrointestinal symptoms, consistent with previous descriptions of OROV infection. However, the possibility of severe outcomes ‒ including neurological or hemorrhagic complications ‒ has been documented, underscoring the importance of continuous monitoring. Although our surveillance did not capture severe cases, this limitation may reflect the sample size rather than the true absence of such presentations. Strengthened surveillance is therefore essential to better characterize the clinical spectrum of OROV, particularly in vulnerable groups such as pregnant women, children, and immunocompromised individuals. Updated reports serve as important alerts for physicians and healthcare professionals when evaluating suspected arbovirus cases, highlighting the need to expand case detection strategies and improve preparedness in both endemic and newly affected regions.

RT-qPCR was the molecular diagnostic method used and proved essential for distinguishing OROV from other acute cases of arboviruses with overlapping symptoms, particularly due the low sensitivity of serological tests and the clinical similarity to dengue, Chikungunya, and Zika that are endemic in the region.^18^^,^^19^ The management of the patients in this report was exclusively symptomatic, hydration, analgesia, and antiemetics, which proved effective and aligns with the current literature, which describes no available specific antiviral therapy or licensed vaccine. Given the potential for neurological complications, severe clinical outcomes and congenital associated malformations, it is essential to promote further research into the pathogenesis of the OROV, improve clinical monitoring protocols, and expand vector prevention and control efforts. In this context, Oropouche fever should be considered an emerging priority on both local and national arbovirus surveillance agendas.

Our study has several limitations. First, it was restricted to a single healthcare center in the city of Salvador, which may limit the generalizability of the findings and laboratory data and longitudinal outcomes are not available. Second, our data were derived exclusively from symptomatic patients; it is possible that a larger number of asymptomatic individuals who did not seek medical care were also affected but went undetected. Third, although the sample size was sufficient to identify transmission among symptomatic cases, it did not allow us to capture severe presentations. Multicenter studies with larger sample sizes, particularly focused on vulnerable populations, will be necessary to better understand the association between OROV infection and severe clinical outcomes. (Table 1).

**Table 1:** Clinical and sociodemographic characteristics of the patients included.

Characteristics	(*n* = 11)
Age (years)	39 (28‒52)
Sex, female	6 (54.5 %)
Day of symptoms	3 (2‒5)
Fever	11 (100 %)
Headache	11 (100 %)
Myalgia	10 (90.9 %)
Retroorbital pain	5 (45.5 %)
Diarrhea	5 (45.5 %)
Nausea	8 (72.7 %)
Arthralgia	6 (54.5 %)
Vomit	3 (27.3 %)
Epigastralgia	1 (9.1 %)
Cough	2 (18.2 %)
Asthenia	2 (18.2 %)
Odynophagia	1 (9.1 %)
Petechiae	4 (36.4 %)
Low back pain	1 (9.1 %)
Exanthema	1 (9.1 %)
Dizziness	0
Conjunctivitis	0
Arthritis	0
Bleeding	0

Characteristics were reported in frequencies and percentages, except age showed in median and interquartile range.

## Conclusions

Oropouche fever is emerging as a relevant arboviral disease in urban areas of Brazil outside the Amazon. The findings presented here reinforce the need to include OROV in the differential diagnosis of acute febrile syndromes, especially during arboviral seasons. Strengthening molecular diagnostics and urban vector surveillance is critical.

## Declarations

Ethics approval and consent to participate: The study was approved by the Comitê de Ética em Pesquisa da Maternidade Climério de Oliveira/UFBA, Brazil (CAAE: 25336819.3.0000.5543/4.691.233, 2021).

Consent for publication: Review.

Availability of data and materials: Data available from the corresponding author upon reasonable request.

## Authors' contributions

AMS conducted data collection and case descriptions. EMN supervised the study, reviewed clinical records, and wrote the final manuscript. All authors read and approved the final version of the manuscript.

Conceptualization: RC and EN. Data curation: RC. Formal analysis: RC, MAG and EN. Methods: RC, CM and EN. Project administration: RC. Resources: Eduardo M. Netto. Software: RC, CM and MAG. Supervision: EN. Validation: RC and CM. Visualization: EN. Writing-original draft: RC. Writing-review & editing: RC, CM, MAG, GS, SS, CB, WKW and EN. All authors had access to all data. All authors contributed to, reviewed, and approved the final version of this manuscript and had final responsibility for the decision to submit for publication.

A Supplementary File containing detailed clinical summaries is submitted alongside this manuscript.

## Funding

RC (88,887.91553/2023–00) are recipient of a Coordination for the Improvement of Higher Education Personnel scholarship (CAPES); the project received funding from CNPq (404193/2019.6) and grants R01AI149502 (WKW) and R01AI186073 (WKW) from the National Institute of Allergy and Infectious Diseases, NIH. The funders were not involved in the conceptualization, data extraction, analysis, preparation of the manuscript and the decision to publish.

## Conflicts of interest

The authors declare no conflicts of interest.

## Appendix Supplementary materials

Image, application 1

## Footnotes

Supplementary material associated with this article can be found, in the online version, at doi:10.1016/j.bjid.2026.105806.
