# Supplementary material for: Oropouche fever cases in Salvador, Bahia, Brazil, 2024
Source: Braz J Infect Dis. 2026 Mar 27;30(2):105806. doi: 10.1016/j.bjid.2026.105806 (PMC13054016; doi:10.1016/j.bjid.2026.105806)
Supplement: Supplementary file 1 [file mmc1.docx]

BJID-D-25-00317

**Supplementary Material 1**

**Molecular arbovirus diagnosis protocol**

Molecular detection was performed using an in-house real-time RT-PCR assay validated by the state public health laboratory.

The one-step singleplex RT-qPCR assay for West Nile virus detection consisted of 8 µL of RNA extracted from serum, 1 µL of BSA (1 ng/µL), 12.5 µL of reaction mix (0.4 mM dNTPs, 6 mM MgSO_4_, 2× buffer), 0.5 µL of each primer, 0.375 µL of probe, and 0.625 µL of RNase-free H_2_O.

The one-step singleplex RT-qPCR assay for Oropouche virus consisted of 8 µL of RNA extracted from serum, 1 µL of BSA (1 ng/µL), 12.5 µL of reaction mix (0.4 mM dNTPs, 6 mM MgSO_4_, 2× buffer), 0.5 µL of each primer, 0.375 µL of probe, and 1.125 µL of RNase-free H_2_O.

The thermal cycling conditions were identical for the tetraplex assay and the singleplex assays for Oropouche and West Nile viruses: 50°C for 10 min, 55°C for 10 min, 94°C for 3 min, followed by 50 cycles of 94°C for 20s and 56°C for 40s. The sequences of primers and probes, as well as the reaction setup and thermocycling conditions for the RT-qPCR assays, are presented below in Table S1 and Figures S1 and S2.

**Table S1** Primers and probes used in the RT‐qPCR assays.

| **Singleplex OroV** | | |
| --- | --- | --- |
| OROV-AS-F01 | GCA CAG AAG CTC TTA AGG TRC ATA G | 0.200 |
| OROV-AS-F02 | CAG AAG CCC TCA AAG TRC ACA G | 0.200 |
| OROV-AS-R01 | CCA GCT TTT MGA YTT ACA CAT CTG | 0.200 |
| OROV-AS-R02 | CCA GCT TTT AGA CTT RCA CAT YTG | 0.200 |
| OROV-AS-P | TexasRed-GGT TAT AAR TCY CTA AGC AAA GC-MGB-NEQ | 0.150 |
| **Singleplex WNV** | | |
| WNV-AS-F01 | GAG ART ATG GAG AGG TGA CAG TGG | 0.200 |
| WNV-AS-F02 | GTG ART ATG GTG AGG TYA CAG TTG | 0.200 |
| WNV-AS-F03 | GAG AAT ATG GAG AAG TRA CRG TGG | 0.200 |
| WNV-AS-R01 | CCA ACA GTC ATC ACR TAG TAT GC | 0.200 |
| WNV-AS-R02 | CCA ACT GAC ATA ACG TAR TAG GC | 0.200 |
| WNV-AS-P | CY5-GTG ARC CAC GST CAG GGA TWG ACA C-BHQ3 | 0.150 |

One-step real-time RT-qPCR reaction and thermocycling conditions.

**Figure S1** Characteristics of RT-qPCR assay. Ten-fold serially diluted OROV cDNA standard.


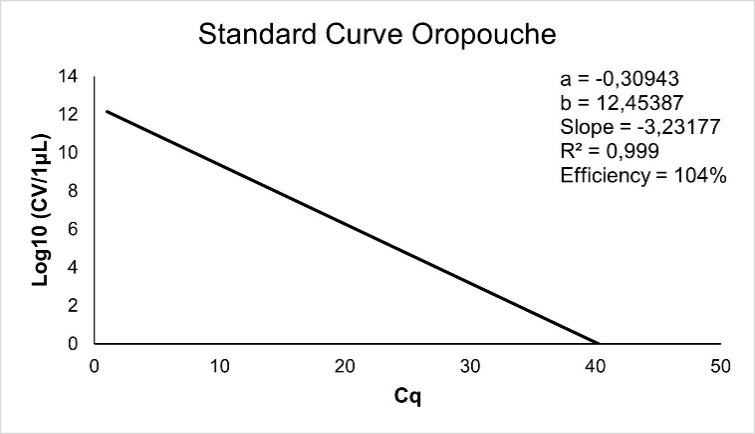


**Figure S2** Characteristics of RT-qPCR assay. Ten-fold serially diluted WNV cDNA standard.


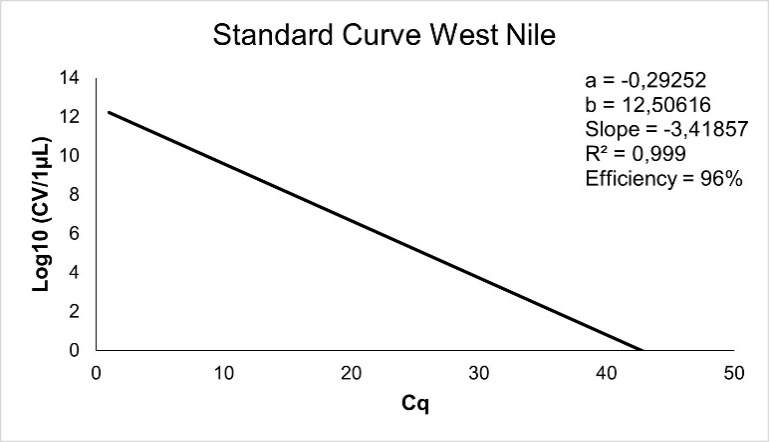


**Supplementary Material 2**

**Clinical summaries**

*Case 1*

A 38-year-old female resident of the Historic Center sanitary district in Salvador, Bahia, sought care at the UPA on July 4, 2024, complaining of headache, fever, arthralgia, sore throat, and fatigue for four days, as well as diarrhea beginning two days earlier. She denied comorbidities, reported an allergy to amoxicillin, and had a complete COVID-19 vaccination schedule.

During triage, she was alert, oriented, with a Glasgow score of 15, blood pressure of 133/83 mmHg, heart rate of 85 bpm, respiratory rate of 18 breaths per minute, temperature of 36.8°C, and oxygen saturation of 99%, reporting mild pain. She was categorized as green priority.

During the medical consultation, Results showed positive IgG and negative IgM for Chikungunya (CHIKV), and negative IgM for Dengue (DENV) and Zika (ZIKV). RT-PCR for Oropouche Fever (OROV) was positive and negative for DENV, ZIKV, CHKV.

Management included symptomatic medication at the facility, prescriptions for home care, and return guidance in case of clinical worsening. Medications prescribed included Metamizole 500 mg (two tablets every 6 hours for pain or fever), Expec syrup (10 mL every 8 hours if coughing), loratadine 10 mg (one tablet per day for five days), and 0.9% saline nasal spray (10 mL per nostril throughout the day, with emphasis on nighttime use). The patient was discharged after symptom improvement.

*Case 2*

A 39-year-old male of mixed race, resident of the Brotas sanitary district in Salvador/BA, sought care at the UPA on April 25, 2024, reporting headache, body aches, and diarrhea that had begun two days prior. He denied comorbidities or allergies. At triage, he was in good general condition, alert, responsive (Glasgow 15), with blood pressure of 145/95 mmHg, heart rate of 80 bpm, temperature of 36.3°C, and oxygen saturation of 100%. On physical examination, he was well-colored, hydrated, afebrile to the touch, non-icteric, non-cyanotic, and hemodynamically stable, without relevant systemic alterations.

Medical management included ordering a complete blood count and rapid Dengue NS1 test, along with symptomatic medications and a medical certificate. Treatment consisted of oral rehydration salts (six sachets), hyoscine with Metamizole (one 500 mg every 6 hours for up to five days if abdominal pain), metoclopramide (one tablet every 8 hours for two days if nausea and vomiting), and Floratil (one tablet every 8 hours for two days). Dietary guidance included avoiding dairy, high-fiber, sugary, and fatty foods, and maintaining fluid intake.

Upon clinical reevaluation, the patient reported complete symptom resolution. Laboratory results showed a normal blood count, Dengue IgG positive and IgM negative, and positive RT-PCR for Oropouche Virus (OROV) and negative for Dengue, Zika, Chikungunya. The patient was discharged with instructions on alarm signs, advised to return if symptoms worsened, and referred for outpatient follow-up at a Primary Health Unit.

*Case 3*

A 30-year-old female of mixed race, resident of the Imbuí sanitary district in Salvador-BA, presented at the UPA on April 28, 2024, reporting insect bites in the past week, along with fever, flu-like symptoms, headache, exanthematous lesions, retro-orbital pain, and diarrhea for the past four days. She denied nausea, vomiting, allergies, or comorbidities.

At triage, she was normotensive (120/80 mmHg), eupneic (20 bpm), febrile (38°C), and tachycardic (117 bpm), with 99% oxygen saturation in room air. On physical exam, she was in good general condition, alert, oriented, well-colored, hydrated, non-icteric, and without systemic abnormalities. Dengue virus infection was considered the primary diagnostic hypothesis.

Initial management included administration of two oral Metamizole tablets, observation, and lab testing.

Test results showed negative rapid Dengue IgG/IgM, and blood count: Hb 14 g/dL, Ht 40.8%, WBC 4,840 mm^3^, platelets 227,000 mm^3^.

The patient showed clinical improvement and was discharged with prescriptions and return precautions. Prescribed medications included Metamizole or paracetamol 500 mg (two tablets every 6 hours orally as needed), oral rehydration salts (10 sachets), and ondansetron 8 mg sublingual (one tablet every 8 hours as needed).

She returned on May 1, 2024, with recurrence of fever, rash, and retro-orbital pain. She denied bleeding, nausea, and vomiting. Rapid Dengue test again returned negative.

Vitals were BP 108/75 mmHg, RR 17 bpm, afebrile (36.6°C), HR 90 bpm, SpO_2_ 99%. Petechial lesions were noted on physical examination.

She was observed again and tested for Dengue, Zika, and Chikungunya ‒ all negative.

She was discharged with a two-day medical certificate and prescription of loratadine 10 mg (one tablet nightly for five days).

*Case 4*

A 30-year-old male, resident of the São Tomé de Paripe district, Salvador/BA, presented at the UPA on May 13, 2024, with three days of fever and myalgia, along with rhinorrhea, sore throat, headache, retro-orbital pain, and nausea. He denied comorbidities or allergies and had received two doses of COVID-19 vaccine.

At triage: mild pain (1–4), normotensive (132/84 mmHg), HR 72 bpm, febrile (38.5°C), eupneic (18 bpm), SpO_2_ 98%. Physical exam: good general condition, responsive, Glasgow 15, well-colored, non-cyanotic, non-icteric, stable, without dehydration. Oroscopy showed pharyngeal hyperemia, Brodsky grade 2, suggestive of bacterial infection.

Management: administration of Benzathine Penicillin 1,200,000 IU IM, hyoscine 4 mg/mL with Metamizole 500 mg/mL, and paracetamol 500 mg. Arbovirus rapid tests were negative, and CBC was unremarkable.

He was discharged with prescriptions: prednisone 20 mg (2 tablets orally once daily for 5 days), Metamizole 500 mg (2 tablets every 6 hours as needed), loratadine 10 mg (1 tablet at night for 5 days), 0.9% saline nasal wash, budesonide spray 64 mcg/dose, and Dexchlorpheniramine maleate (2 mg/5 mL) + Betamethasone (0.25 mg/5 mL) syrup (10 mL every 8 hours).

*Case 5*

A 53-year-old black male, resident of Barra/Rio Vermelho district in Salvador/BA, presented on April 24, 2024, with diarrhea (>5 episodes, no blood), vomiting (4 episodes, no blood), myalgia, and frontal headache for two days. History of hypertension treated with losartan, amlodipine, atenolol, and hydrochlorothiazide. Known allergy to benzathine penicillin.

Triage: BP 180/88 mmHg, HR 80 bpm, Temp 36°C, RR 20, SpO_2_ 100%. Physical exam: good condition, alert, oriented, hydrated, without systemic abnormalities. He received symptomatic medication and was discharged with home prescriptions: Metamizole, hyoscine, metoclopramide, oral rehydration salts, and *Saccharomyces boulardii* capsules 200 mg, plus return instructions.

Returned on April 25 with persistent headache (with retro-orbital pain), myalgia, nausea, vomiting, diarrhea, and fever for 2 days. Physical exam: good condition, Glasgow 15, hydrated, afebrile, well-colored, no systemic abnormalities. Vitals: BP 172/105 mmHg, HR 60 bpm, Temp 36°C, SpO_2_ 99%.

He received ondansetron, hyoscine, clonidine, and Metamizole with significant symptom improvement after 40 minutes. Hemogram was normal. Dengue IgG positive, IgM negative. RT-PCR positive for Oropouche Virus (OROV). He was discharged with outpatient management instructions and prescriptions.

*Case 6*

A 32-year-old female, resident of the Barris district in Salvador, Bahia, sought care at the UPA on April 18, 2024, presenting with myalgia, fever, headache, lower back pain, and nausea for five days. She had been self-medicating with Metamizole at home. During history taking, she reported not having been tested for Dengue. She had a known diagnosis of asthma but denied allergies or flu-like symptoms.

At triage, she was normotensive (120/80 mmHg), normocardic (80 bpm), afebrile (36°C), eupneic (20 breaths per minute), and with oxygen saturation of 100% on room air. On physical examination, she was in good general condition, alert and oriented, Glasgow 15, hydrated, non-icteric, afebrile, eupneic, and without abnormalities in other systems.

Chest X-Ray, Dengue NS1, and rapid test were requested. Medications administered at the UPA included omeprazole 20 mg, Metamizole 500 mg, and hyoscine 20 mg/mL for symptom relief. Test results showed positive IgG and negative IgM for Dengue, and positive RT-PCR for Oropouche Virus (OROV).

After clinical improvement, the patient was discharged with guidance on alarm signs and return instructions prescriptions for home use, including salbutamol spray 100 mcg, oral rehydration salts, Metamizole 500 mg, and ondansetron 8 mg.

*Case 7*

A 23-year-old Black female, resident of the Itapuã district, Salvador/BA, presented on April 2, 2024, returning of Valença/BA, with sudden onset (1-day prior) of supraorbital headache, self-reported fever, abdominal pain, nausea, diarrhea, sweating, and myalgia for a period of one week. She denied comorbidities or allergies.

On examination, she was in good general condition, alert, Glasgow 15, well-colored, hydrated, afebrile to the touch, and hemodynamically stable. Vital signs: axillary temperature 38.5°C, BP 110/72 mmHg, HR 115 bpm, RR 18 breaths/min, SpO_2_ 98%.

NS1 rapid tests for DENV, ZIKV and CHKV were negative. She received Metamizole and paracetamol in the unit for symptom relief. Lab results showed mildly reduced red blood cell count (3.89 million/mm^3^) and hematocrit (35.9%), with other parameters normal. RT-PCR was positive for OROV and negative DENV, ZIKV and CHKV.

After clinical reassessment, she was discharged with prescriptions for oral rehydration salts, Metamizole 500 mg (2 tablets every 6 hours if pain or fever), and ondansetron 8 mg (1 tablet every 8 hours if nausea or vomiting).

*Case 8*

A 58-year-old Black female, resident of the Barra/Rio Vermelho district, Salvador/BA, presented to the UPA on April 18, 2024, reporting malaise, myalgia, and headache starting four days earlier, along with sudden onset of generalized pruritus 6 hours before. She had a prior diagnosis of systemic arterial hypertension, controlled with losartan, and denied drug allergies.

At triage, she was lucid, oriented, in good general condition, with BP 165/104 mmHg, HR 63 bpm, RR 17 breaths/min, temperature 36.5°C, SpO_2_ 99%, and reported mild pain. On physical examination, she was well-colored, hydrated, non-icteric, non-cyanotic, and hemodynamically stable.

Symptomatic medication (Metamizole and dexamethasone) was administered at the facility, along with guidance on warning signs and prescriptions for home use: oral rehydration salts and analgesics (Metamizole or paracetamol 500 mg as needed).

RT-PCR results was positive of OROV and negative for DENV, ZIKV and CHKV.

*Case 9*

A 52-year-old male of mixed race, resident of the Historic Center district in Salvador/BA, presented at the UPA on April 28, 2024, at 5:00 p.m., with complaints of nightly fever, chills, neck stiffness, right upper limb edema, myalgia, chest arthralgia, headache, dental arch stiffness, and productive cough with chest discomfort during coughing, beginning five days prior. He reported a history of systemic hypertension and glaucoma, treated with losartan. He denied medication allergies.

At triage, he had BP 188/121 mmHg, HR 101 bpm, temperature 36°C, RR 20 breaths/min, and SpO_2_ 97%. He was referred for medical evaluation, received symptomatic medication, and underwent laboratory tests, rapid arbovirus testing, and chest X-Ray.

Lab results showed normal blood count, urea, and creatinine. Serologies were negative for COVID-19, Dengue, Chikungunya, and Zika, and positive for Oropouche Virus (OROV).

At 8:00 p.m., the patient was reassessed after receiving clonidine and codeine and presented complete symptom resolution with stable vital signs. He was discharged with prescriptions for home use: amoxicillin, nimesulide, Metamizole, acetylcysteine, saline solution, and budesonide. He was advised on warning signs and referred for outpatient follow-up.

*Case 10*

A 21-year-old male of mixed race, resident of the Historic Center district, Salvador/BA, presented to the UPA on May 19, 2024, at 11:00 a.m., with complaints of headache, retro-orbital and dental arch pain, nausea, vomiting, myalgia, arthralgia, skin rash, and fever episodes starting the previous day. He denied comorbidities or allergies.

At triage, he was alert, oriented (Glasgow 15), with BP 144/97 mmHg, HR 122 bpm, RR 18 breaths/min, temperature 39°C, and SpO₂ 100%.

During the medical evaluation, he was in good general condition. Medications administered included codeine with paracetamol, ondansetron, Metamizole, and IV hydration with 500 mL of 0.9% saline. A clinical reassessment was scheduled.

Lab results showed a normal blood count and a positive result for Oropouche Virus (OROV) andnegative for Zika, Dengue, Chikungunya.

At 2:00 p.m., he was reassessed and showed complete clinical improvement. He was discharged with pharmacological prescriptions for continued treatment at home, warning sign guidance, and instructions to return if symptoms worsened.

*Case 11*

A 39-year-old Peruvian woman presented to the UPA on June 17, 2024, at 10:00 a.m. with complaints of fever, headache, nausea, arthralgia, and a rash on her neck, hands, and armsstarting 7-days earlier. The patient also reported that, while living in Salvador, Brazil, she was tested positive for Zika virus (2015) and Chikungunya virus (2020). She had traveled to Peru and Mexico in the previous two months and had resided in the United States last year; she has residing in Brazil since April 2024.

During the evaluation, a complete blood count showed: hemoglobin 12.2 g/dL, microcytosis (+) and hypochromia (+), PCR: 2.2 mg/L, and positive result for OROV and negative for Zika, Dengue, Chikungunya. She received paracetamol and intravenous hydration. After a reassessment, she was discharged with instructions to return if symptoms worsened. Clinical evaluation one week later included a decrease in the rash and arthralgia. She reported no other symptoms. No medication was prescribed.
